# Carbon Nanoelectrodes for the Electrochemical Detection of Neurotransmitters

**DOI:** 10.1155/2018/3679627

**Published:** 2018-08-01

**Authors:** Alexander G. Zestos

**Affiliations:** Department of Chemistry, Center for Behavioral Neuroscience, American University, Washington, DC 20016, USA

## Abstract

Carbon-based electrodes have been developed for the detection of neurotransmitters over the past 30 years using voltammetry and amperometry. The traditional electrode for neurotransmitter detection is the carbon fiber microelectrode (CFME). The carbon-based electrode is suitable for *in vivo* neurotransmitter detection due to the fact that it is biocompatible and relatively small in surface area. The advent of nanoscale electrodes is in high demand due to smaller surface areas required to target specific brain regions that are also minimally invasive and cause relatively low tissue damage when implanted into living organisms. Carbon nanotubes (CNTs), carbon nanofibers, carbon nanospikes, and carbon nanopetals among others have all been utilized for this purpose. Novel electrode materials have also required novel insulations such as glass, epoxy, and polyimide coated fused silica capillaries for their construction and usage. Recent research developments have yielded a wide array of carbon nanoelectrodes with superior properties and performances in comparison to traditional electrode materials. These electrodes have thoroughly enhanced neurotransmitter detection allowing for the sensing of biological compounds at lower limits of detection, fast temporal resolution, and without surface fouling. This will allow for greater understanding of several neurological disease states based on the detection of neurotransmitters.

## 1. Introduction

Dopamine is an important neurotransmitter which functions in reward, cognition, voluntary movement, addiction, and learning [1–5]. The detection of dopamine and other neurotransmitters is important for studying diseases such as Parkinson’s disease, drug abuse, and depression [6]. Traditionally, glass insulated carbon fiber microelectrodes (CFMEs) have been used to detect neurotransmitter concentrations such as dopamine in biological samples [7–11]. *In vivo* voltammetry has been instrumental for the detection of neurotransmitters and monitoring of neurotransmission in the brain especially in response to certain receptor agonist or antagonist drugs, which could allow us to understand more about human disease states such as Parkinson’s disease [12–14]. This review seeks to explore novel methods of electrode fabrication for neurotransmitter detection. Novel microelectrodes are being explored due to their possible usage as chronic sensors [15], increased sensitivity [16], resistance to surface fouling [17], and faster electron transfer kinetics with respect to carbon fiber microelectrodes [16]. This review will examine novel methods of developing electrode materials. Both alternative insulations and alternative electrode materials will be examined (carbon nanotube fibers and metal wires). Superior nanoelectrode technology through novel methods of construction will be discussed.

This review aims to discuss novel insulations for carbon nanoelectrodes and different carbon nanoelectrode materials and their performance in neurotransmitter detection. Alternative insulations including epoxy insulation have been chosen to replace the glass capillary insulation. Glass has been used for over twenty years; however, it has certain drawbacks such as breaking *in vivo*, which is why it cannot be used for testing in higher order primates or for long-term chronic measurements. Carbon nanotube (CNT) fibers have been epoxy insulated to create carbon nanotube fiber microelectrodes through this novel electrode development procedure. Polyethyleneimine (PEI) carbon nanotube fiber microelectrodes have been created using the epoxy insulation. They have displayed superior characteristics to poly(vinyl alcohol) (PVA) [18] carbon nanotube fiber microelectrodes such as higher sensitivities, faster electron transfer kinetics, and a resistance to surface fouling by serotonin and 5-hydroxyindoleacetic acid (5-HIAA). The same epoxy insulation was used to form acid spun carbon nanotube fiber microelectrodes. Acid spun CNT electrodes were dissolved in chlorosulfonic acid without the use of sonication, surfactants, or polymer coatings that would serve as impurities. They are then syringed into water or acetone, which displaced the acid to form vertically aligned CNT fibers. Along with PEI-CNT fibers and CNT yarns, they have displayed a sensitivity towards dopamine that is independent of the wave application frequency. This could be useful in detecting dopamine at frequencies at which neurotransmission actually occurs. Carbon nanospikes have also been grown onto metal wire electrodes for the detection of neurotransmitters. The idea of this study was to coat a substrate with carbon nanotubes that is more conductive than the carbon fiber to not slow down electron transfer. This method has been used to form a wide variety of metal electrodes. The carbon coating method creates novel electrode materials out of metals that were previously unable to detect dopamine before carbon coating.

## 2. Electrochemistry/Electrode Fabrication

### 2.1. Fast Scan Cyclic Voltammetry for Neurotransmitter Detection *In Vivo*.

**Figure 1:**
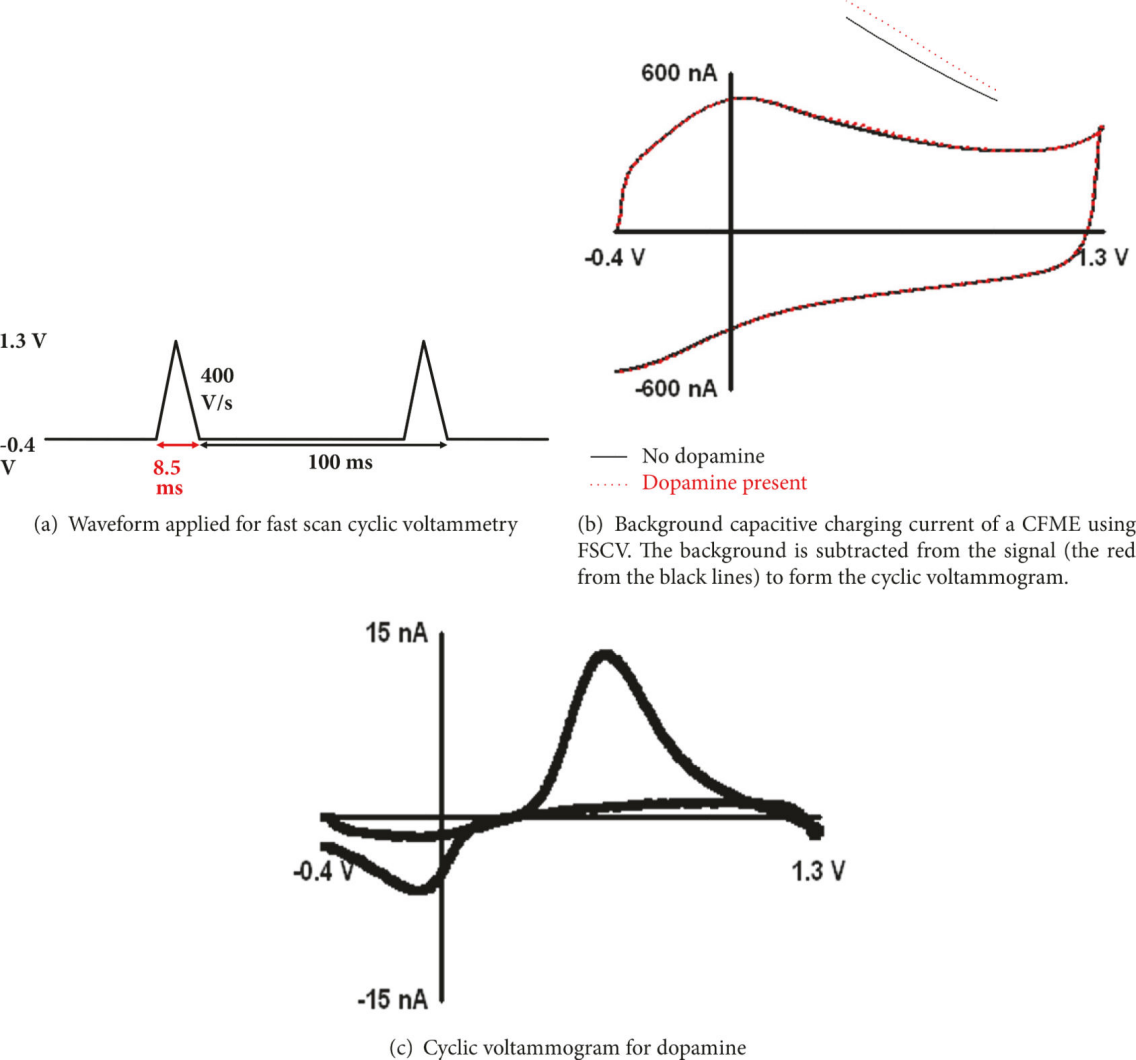
Figures provided from Venton Lab.

Fast scan cyclic voltammetry (FSCV) is an electroanalytical technique that can be used to measure neurotransmitter concentrations *in vivo* [20]. A carbon fiber (typically 7 *μ*m in diameter) is used as the working electrode, while the reference electrode is often an Ag/AgCl wire (.197 V). A commonly used waveform for dopamine detection ramps the voltage linearly from the holding potential (−0.4 V) to the switching potential (1.3 V) and back at a scan rate of 400 V/sec to oxidize and, subsequently, reduce the analyte (Figure 1(a)). The background charging current (Figure 1(b)) is a function of the scan rate and electroactive surface area of the electrode. Background subtraction reveals the cyclic voltammogram (CV) of the analyte being detected. In Figure 1(b), the black line is subtracted from the red line to give the background subtracted CV (Figure 1(c)) for dopamine. Dopamine is oxidized to dopamine-o-quinone (DOQ) and DOQ is reduced back to dopamine [21]. The shape and position of the cyclic voltammogram are a molecular fingerprint for the specific molecule being detected. FSCV has been used for the detection of neurotransmitters in drosophila melanogaster [22], rats [23], brain slices [10], and primates such as monkeys [24] and humans [25]. The interaction of dopamine with the surface of the carbon fiber is adsorption controlled partly because its amine group is positively charged (protonated) at physiological pH [21]. It undergoes an electrostatic interaction with the negatively charged oxides at the surface of the carbon fiber in addition to hydrogen bonding and dipole-dipole interactions [26]. Current is directly proportional to both concentration and scan rate for adsorption controlled processes.

### 2.2. Glass Insulated Carbon Fiber Microelectrodes.

**Figure 2:**
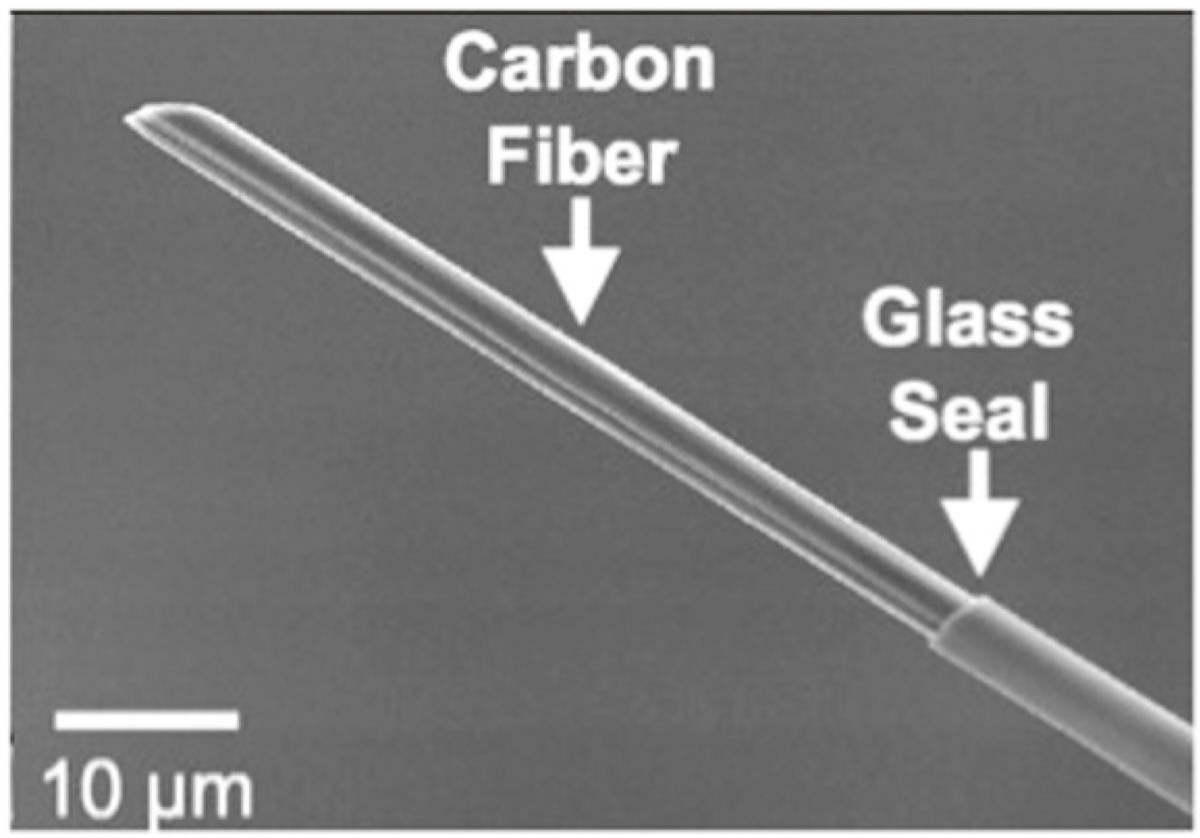
Scanning electron micrograph (SEM) image of a glass capillary insulated CFME (figures provided from Venton Lab). Figure comes from [27].

Carbon fiber microelectrodes have been utilized for the detection of neurotransmitters *in vivo* [8]. The electrodes are traditionally insulated in glass capillary tubes pulled to a fine taper using a vertical capillary puller [12, 34–37]. The carbon fiber protrudes through the tapered end of the glass capillary and is usually cut to 50–100 microns as shown in Figure 2. The electrode is then backfilled with potassium chloride to create an electrical connection. Although glass insulations have been used for over twenty years and are the standard for neurochemical detection, they have certain drawbacks. First, alternative nanomaterials such as carbon nanotube fibers cannot be insulated with glass capillary insulation [38]. Vertical capillary pullers cannot be used with carbon nanotube fibers/yarns because of the altered tensile strengths and thicknesses. Second, glass insulations are currently not allowed for *in vivo* testing of non-human primates such as monkeys because of possible shattering or breakage in tissue [24]. Epoxy insulation provides alternative insulation to glass that does not require the use of a vertical capillary puller [39].

### 2.3. Alternative Insulations for Electrode Materials.

Novel electrode construction techniques have been examined for many years. The Baker group has studied the pyrolysis of parylene C to coat conical quartz electrodes to create electrodes with high thermal stabilities [40]. The chemical vapor deposition and subsequent pyrolysis of parylene C created a very conductive substrate that can be used for electrochemical detection. The subsequent vapor deposition of parylene provides the insulation, while masking in polydimethylsiloxane (PDMS) during vapor deposition preserves the electroactive region. Another quartz electrode involves the pyrolysis of methane inside of pulled quartz capillaries [41]. The resulting pyrolytic carbon forms a film on the inside of the capillary resulting in microring geometry. These electrodes can detect 1 *μ*M dopamine.

In the past, many alternative insulations have been examined in addition to glass insulations for carbon fiber microelectrodes [42]. Poly(oxyphenylene) and a copolymer of 2-allylphenol and phenol were electropolymerized onto the surface of the carbon fiber upon applying a positive potential [42, 43]. The polymers provided a thin insulation on the carbon fiber; however, the fibers must be masked using a compound such as paraffin wax in order to preserve the electroactive area of the electrode (the noninsulated region). The removal of the masking agent in addition to the lack of robustness of the thin and flimsy (nm thick) insulation provides many experimental challenges. Varying polymer concentrations, times of electrodeposition, and pH provide insulations of variable thicknesses that are not easily reproducible.

Another insulation for carbon fiber microelectrodes involves the anodic electrophoretic deposition of paint onto the surface of carbon fiber to serve as the insulator [44, 45]. The anodic paint consists of an aqueous dispersion of poly(acrylic carboxylic acid), Glassphor ZQ 84–3211, and resins of micellar structure. The negatively charged water soluble portion of the polymer has carboxylate end groups that are neutralized by acidification that leads to the precipitation of micelles. These negatively charged micelles are electrophoretically attracted to the anode. Water hydrolysis produces protons that precipitate the micelles as a thin, uniform, and tightly adhered polymer film on the electrode surface. Compared to the electrodeposition of polymers containing phenolic compounds, the insulation of carbon fibers by anodic electrophoretic deposition is much simpler, has a shorter processing time, and is less toxic.

### 2.4. Insulations for Metal Electrodes.

The use of epoxy insulations for electrodes has been examined by the Wightman group where conical epoxylite insulated Tungsten wire tips were used as substrates for the preparation of platinum and gold ultramicroelectrodes (UMEs) [46]. Gold and platinum were electroplated onto the surface of the Tungsten and then coated with a photoresist followed by pyrolysis. Epoxylite epoxy was used to coat the entire electrode except for the tip, which provided an exposed carbon surface able to perform voltammetric measurements. These novel UMEs had similar electrochemical behavior to electrodes made from wires or carbon fibers insulated with glass capillaries [46]. Selimovic et al. created a new method to form epoxy embedded macroelectrodes to integrate electrochemical detection with microchip-based analysis systems [47]. The use of a Teflon mold was ideal because of its nonstick properties, which allows for the curing of epoxy onto the surface of electrodes, and their subsequent removal from the mold.

A development of a novel epoxy insulation for carbon fiber and carbon nanotube fiber microelectrodes was also achieved [39]. The use of a nonstick Teflon mold will be used as a substrate to coat the electrode materials. Silver epoxy was used to connect one protruding end to a gold pin that fits into the head stage of the potentiostat to form an electrode. This novel electrode development facilitated rapid testing of novel electrode materials including carbon nanotube fiber electrodes and metal electrodes coated in carbonaceous materials.

### 2.5. Carbon Nanotubes

#### 2.5.1. Carbon Nanotubes Introduction.

**Figure 3:**
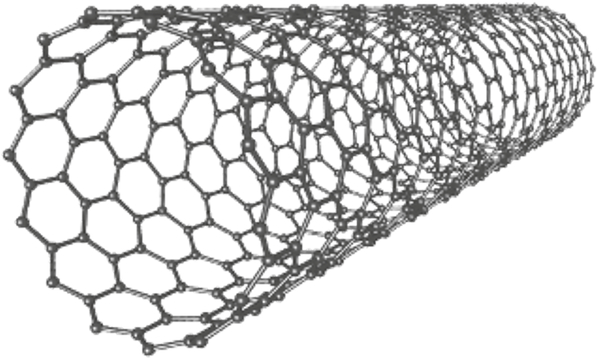
Structure of single wall carbon nanotube (SWCNT).

Iijima discovered carbon nanotubes in 1991 [48]. The novel carbon nanotubes were produced in a similar fashion to C_60_ and other fullerenes. Iijima utilized arc discharge synthesis where needles grew at the negative end of the electrode used for discharge. The carbon-atom hexagons were arranged in a helical fashion about the needle axis in diameters ranging from 4 to 30 nm and up to 1 mm in length. The nanotubes resembled layers of graphite sheets rolled up with respect to the needle axis. Iijima and coworkers analyzed electron diffraction patterns of the newly created carbon nanotubes to verify their hexagonal orientation and highly sp^2^ hybridized chemical structure. This highly sp^2^ hybridized structure gives the nanotubes superior mechanical properties and very high conductivity with respect to other carbon-based materials. Iijima and coworkers further characterized carbon nanotubes by discovering single wall carbon nanotubes (SWCNTs) (see Figure 3) that form in the gas phase as opposed to the MWCNTs that grow on the carbon cathode during carbon arc synthesis [49]. The authors used iron as a homogeneous catalyst in the vapor phase that assisted in the formation of shelled tubules. The mirror symmetry in diffraction patterns confirmed the presence of single shelled tubules. CNTs have thermal stability up to 1400°C in a vacuum oven and gas adsorption properties such as to hydrogen and electrical superconductivity [50].

### 2.5.2. Carbon Nanotube Based Electrochemical Sensors.

Carbon nanotube based electrodes have been used for the detection of neurotransmitters [16]. The high conductivity of CNTs makes them attractive as electrode sensing materials [51]. CNT electrodes have been utilized as detectors for neurotransmitters/ neurochemicals [52], proteins [53], and DNA [54] and as enzyme sensors [55]. CNT electrodes can detect proteins through direct and indirect electrochemistry, chemical reactions, and through the gating of FET (field emission transistors) [53]. Wang’s group designed a novel CNT-based electrode for direct amperometric and voltammetric determination of insulin [56]. Other proteins detected were streptavidin and albumin [57]. CNT-based electrodes have also been used as enzyme sensors to detect sugars such as glucose and galactose [58–61] in addition to other biological compounds such as cholesterol [62] and DNA [63, 64]. The addition of CNTs to enzyme sensors further facilitates the tunneling of electrons through the delocalization of electrons due to sp^2^ hybridization. They also have electrocatalytic effects for hydrogen peroxide or glucose detection on their edge plane sites [65].

#### 2.5.3. Carbon Nanotube Based Detection of Dopamine.

Britto et al. developed the first carbon nanotube paste electrode for the detection of dopamine using voltammetry [66]. MWCNTs were blended with a binder such as bromoform to create a CNT paste. The paste was packed into a glass capillary to form a carbon nanotube paste electrode. Cyclic voltammetry (CV) and differential pulse voltammetry (DPV) were both used to study the oxidation of dopamine. As opposed to other carbon-based electrodes, a perfect/ideal Nernstian reversibility (~30mV) peak separation was seen for CNT paste electrodes, which was not expected from carbon-based electrodes most likely due to the higher conductivity of the carbon nanotubes. Furthermore, no decrease in sensitivity was seen when the electrode was implanted into goat brain tissue. Further work from the same group saw improved charged transfer at carbon nanotube electrodes for the electrocatalytic reduction of dissolved oxygen, which is important for fuel cells [67].

### 2.6. Dip-Coating CNTs onto Carbon Fibers for Neurotransmitter Detection.

**Figure 4:**
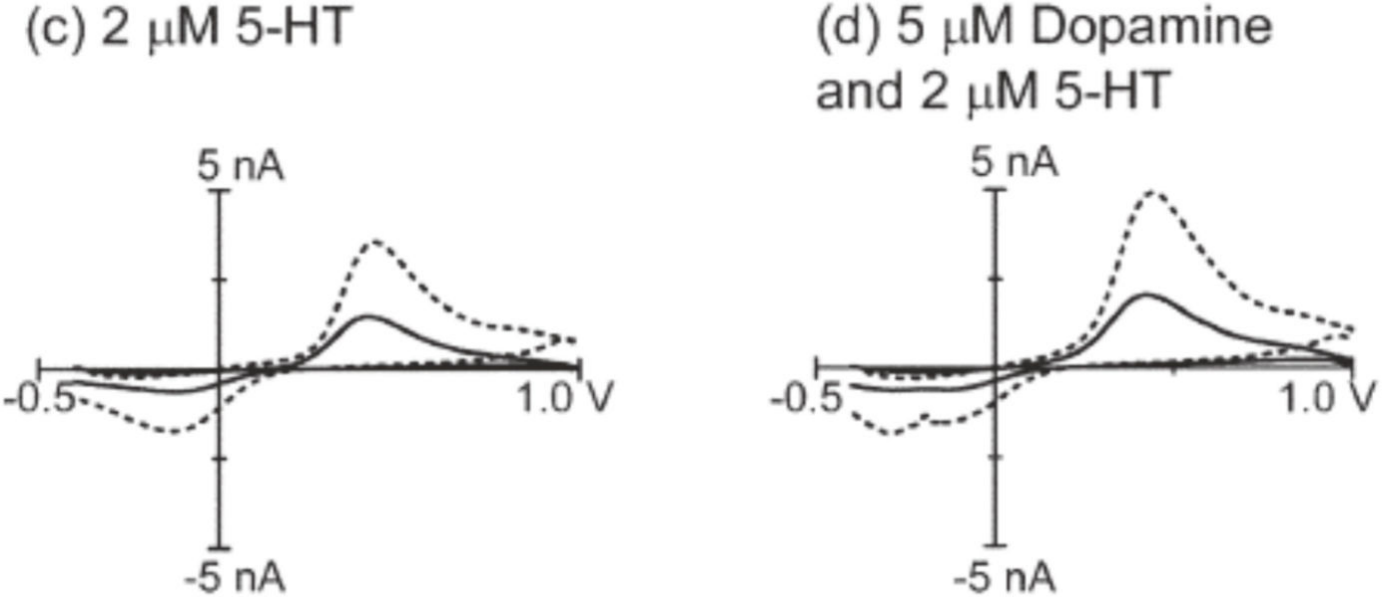
Comparison between bare (solid line) and CNT coated (dashed line) disk electrodes. (c) Background subtracted CVs for 2 *μ*M serotonin. (d) Background subtracted CVs for 2 *μ*M serotonin and 5 *μ*M dopamine [17]. Reprinted from [17].

The Venton group has developed several strategies for making carbon nanotube modified electrodes for neurotransmitter detection [16, 38]. Carbon nanotubes increase sensitivity and have faster electron transfer kinetics. Dip-coating a carbon fiber microelectrode in a suspension of carbon nanotubes in an organic solvent such as dimethylformamide (DMF) results in a more sensitive electrode [17]. However, this dip-coating procedure is not reproducible and decreases signal to noise ratios (S/N) [17]. Venton and Swamy saw 2–6-fold increases in sensitivity in dopamine and serotonin detection using fast scan cyclic voltammetry with respect to disk electrodes without sacrificing temporal resolution as other pretreatments have been known to do (Figure 4) [17]. The increase in sensitivity was likely a result of the increase in the number of adsorption sites because of the increase in the surface area by carbon nanotube modifications. The carbon nanotubes also increased electron transfer kinetics at relatively lower switching potentials and slower scan rates, which did not result in the overoxidation of the surface of the carbon fiber.

The carbon nanotube coatings also aided in codetecting serotonin and dopamine [17]. These two neurotransmitters both oxidize at 600 mV, and their reduction potentials differ by approximately only 200 mV with FSCV [17]. A special waveform applied for serotonin detection that reduces fouling does not present the reduction peak of dopamine, which makes it difficult to codetect both molecules. Serotonin produces hydroxylated dimers and oxidation products that irreversibly adsorb to the surface of the carbon fiber, which block sites for further adsorption [17]. Disk carbon fiber electrodes lost approximately 40% of their sensitivity over time, while carbon nanotube modified carbon fiber microelectrodes (CFMEs) lost less than 10% of their signal. Oxidative by-products of serotonin do adsorb to the CNT surface, but do not inhibit electron transfer. This means that the surface is not as easily passivated after CNT dip-coating [17].

The mechanism of the resistance to fouling to CNT materials is not completely understood. It has been shown that the extent of fouling is dependent on applied waveform; however, not all CNT materials are antifouling, as shown by the Mailperson group [68]. Studies of thin carbon films have found that adding oxide groups while maintaining a high sp^2^ conjugation also helps with resistance to serotonin fouling [69]. PVA-CNT fiber microelectrodes were also resistant to fouling by large concentrations of dopamine and CNTs were found to resist the first phase of fouling, the growth of the insulating layer from the polymerization products [70]. Resistance to fouling at CNT ends is often attributed to the higher density of edge plane sites that are more prevalent in CNT materials. Indeed, some studies of edge plane pyrolytic graphite electrodes have found that they have similar antifouling properties to CNT-based electrodes [71]. Antifouling properties of PEI-CNT fiber microelectrodes have also been created. PEI-CNT fiber microelectrodes were found to be antifouling with respect to physiological concentrations of both serotonin and 5-hydroxyindoleacetic acid (5-HIAA).

### 2.7. Effect of Functional Groups on the Sensitivity of CNT Modified CFMEs.

Jacobs et al. continued this work by dip-coating carbon fiber microelectrodes with CNTs functionalized with carboxylic acid, amides, and octadecylamine [16]. Dip-coating CFMEs with carboxylic acid and amide functionalized CNTs increased sensitivity 2–6-fold; however, dip-coating with octadecyl amine displayed no increase in sensitivity because of increased electrostatic interactions between the positively charged (cationic) species such as dopamine and the negatively charged carboxylic groups. On the other hand, the positively charged octadecylamine repelled the positively charged dopamine. Similarly, the electron transfer kinetics were much faster for the amide and carboxylic CNT coatings rather than the octadecyl amine CNT coatings. The long and bulky alkyl chain (18 Cs) was thought to sterically hinder electron transfer, therefore, significantly decreasing conductivity.

### 2.8. Nafion-CNT Coatings on Carbon Fibers.

Nafion was also used to coat carbon fiber microelectrodes along with CNTs [72]. Nafion is a cation exchange polymer with a negative charge that is used to attract cationic species and repel anionic species (ATP or ascorbic acid). Dip-coating in CNT-Nafion suspensions increased sensitivity (peak oxidative current) 4-fold compared to bare electrodes and 2-fold compared to Nafion coated electrodes, and the electrodes were six times as sensitive to adenosine over ATP. This can be explained by the fact that ATP is a negatively charged molecule (from the triphosphate groups), which undergoes an electrostatic repulsion with respect to the negatively charged Nafion. On the other hand, the neutrally charged adenosine is not repulsed by the Nafion.

The aforementioned Nafion-CNT coatings have been thoroughly compared to overoxidized polypyrrole- (oPPY-) CNT coatings [73]. Both types of electrodes were 3–4-fold more sensitive to dopamine than bare CFMEs without sacrificing temporal resolution or time response. Polypyrrole was electropolymerized and overoxidized onto the surface of the carbon fiber to form oPPY-CNT coatings. OPPY-coatings had faster electron transfer kinetics and more sensitivity *in vivo* and maintained selectivity over anions. The reason for this is that Nafion is known to wrap nanotubes, which makes it electrostatically repel anions from the CNTs in the Nafion-CNT coating.

However, the deposition of Nafion onto the surface of CFMEs is not only achieved by dip-coating. Hashemi et al. electrodeposited Nafion onto the surface of CFMEs [74]. Nafion was electrodeposited onto the surface of CFMEs at a potential of 4.0 V. The author electrodeposited Nafion onto the surface of CFMEs, so that they would deposit a thin and uniform layer of Nafion. They did this primarily to electrostatically repel the metabolite interferant 5-hydroxyindole acetic acid (5-HIAA) from the surface of CFMEs during the *in vivo* detection of serotonin. 5-HIAA is a breakdown product of serotonin that has physiological concentrations ten-times greater than that of serotonin. 5-HIAA is repelled by the Nafion because it is negatively charged, while serotonin is not. However, the electrodeposition of Nafion on the surface makes electrodes highly absorptive, which can delay time responses *in vivo*.

The Andrews group also compared the antifouling properties of Nafion in comparison to base-hydrolyzed cellulose acetate (BCA) and fibronectin [75]. CFMEs immersed in brain tissue were tested with the three coatings. BCA was found to be relatively fouling resistant. Fibronectin coatings were fouled significantly exhibiting moderate losses in sensitivity. On the other hand, Nafion coatings (without CNTs) increased sensitivity for both dopamine and norepinephrine, but not for serotonin. Substantial fouling occurred in CFMEs that were both electrodeposited and dip-coated with Nafion, comparable to bare CFMEs. Thus, this supports the hypothesis that CNTs could play a role in the antifouling process.

### 2.9. Vertically Aligned CNT Forests Grown on CFMEs.

Methods have been developed to grow vertically aligned CNT forests on the surface of a carbon fiber using a chemical self-assembly method [28]. Evenly dip-coating CNTs onto a carbon fiber is difficult because a dense and uniform layer of CNTs is hard to deposit on the electrode surface. Usually, large CNT aggregates are formed, which creates low reproducibility and much noise. In this study, carbon fibers were coated first in an iron-hydroxide decorated Nafion film and then in a suspension of short carboxylic acid functionalized CNTs suspended in dimethylformamide (DMF). A polished disk carbon fiber microelectrode was used to have an even and uniform surface for CNT forest self-assembly. The self-assembly method caused the CNTs to stand on the ends, which is efficacious because the ends of CNTs are likely to be the best sites for electron transfer [28].

**Figure 5:**
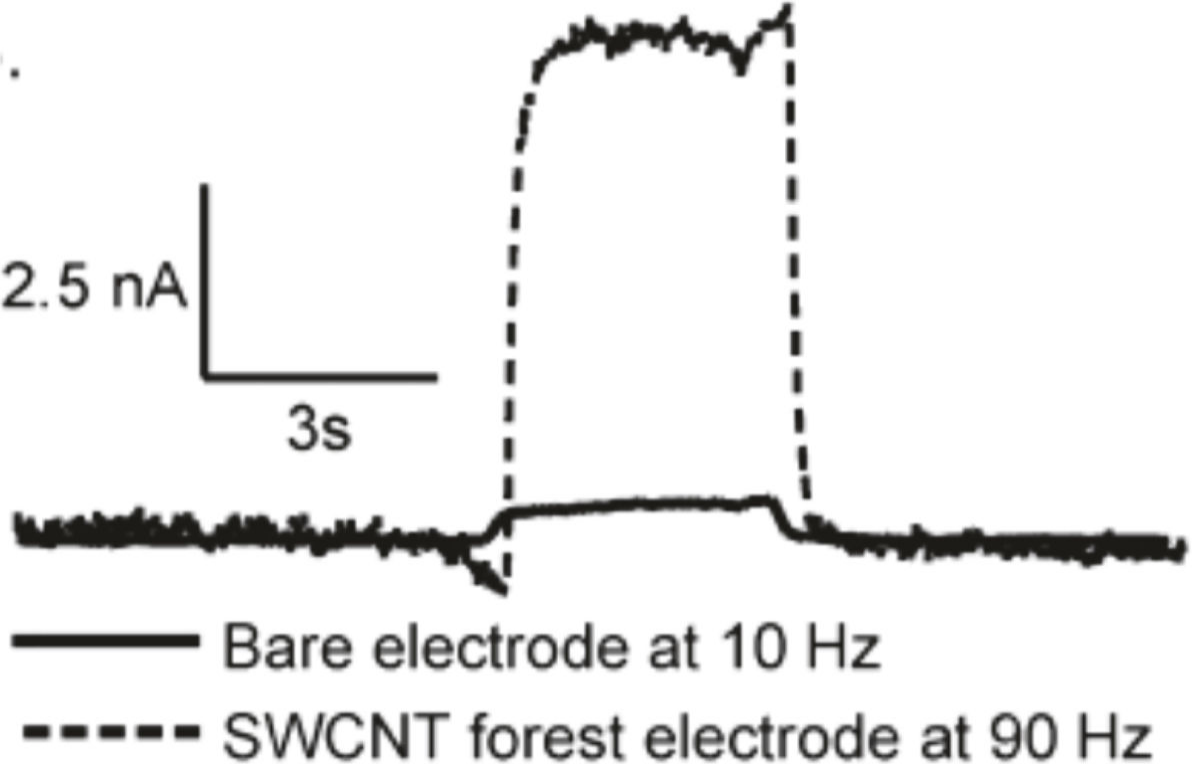
Current versus time trace depicting 40-fold increase in sensitivity after dipping in CNT suspension to form CNT forest electrode [28]. Reprinted from [28].

The authors saw a remarkable 30–40-fold increase sensitivity upon applying the self-assembled coating without such a marked increase in the background current. The CNT forest coated electrodes had the same sensitivity as disk uncoated carbon fibers at 10 Hz upon increasing the wave application frequency to 90 Hz which allowed the authors to scan at much higher frequencies without compromising sensitivity (see Figure 5). Six other analytes increased in sensitivity at vertically aligned carbon nanotube electrodes. The electrode was also used to measure endogenous dopamine changes in the ventral nerve cord (VNC) of *Drosophila*. The SWCNT forest electrodes actually detected higher current for dopamine at 90 Hz than the bare electrodes could detect at 10 Hz.

### 2.10. Wet Spinning Carbon Nanotube Fibers.

**Figure 6:**
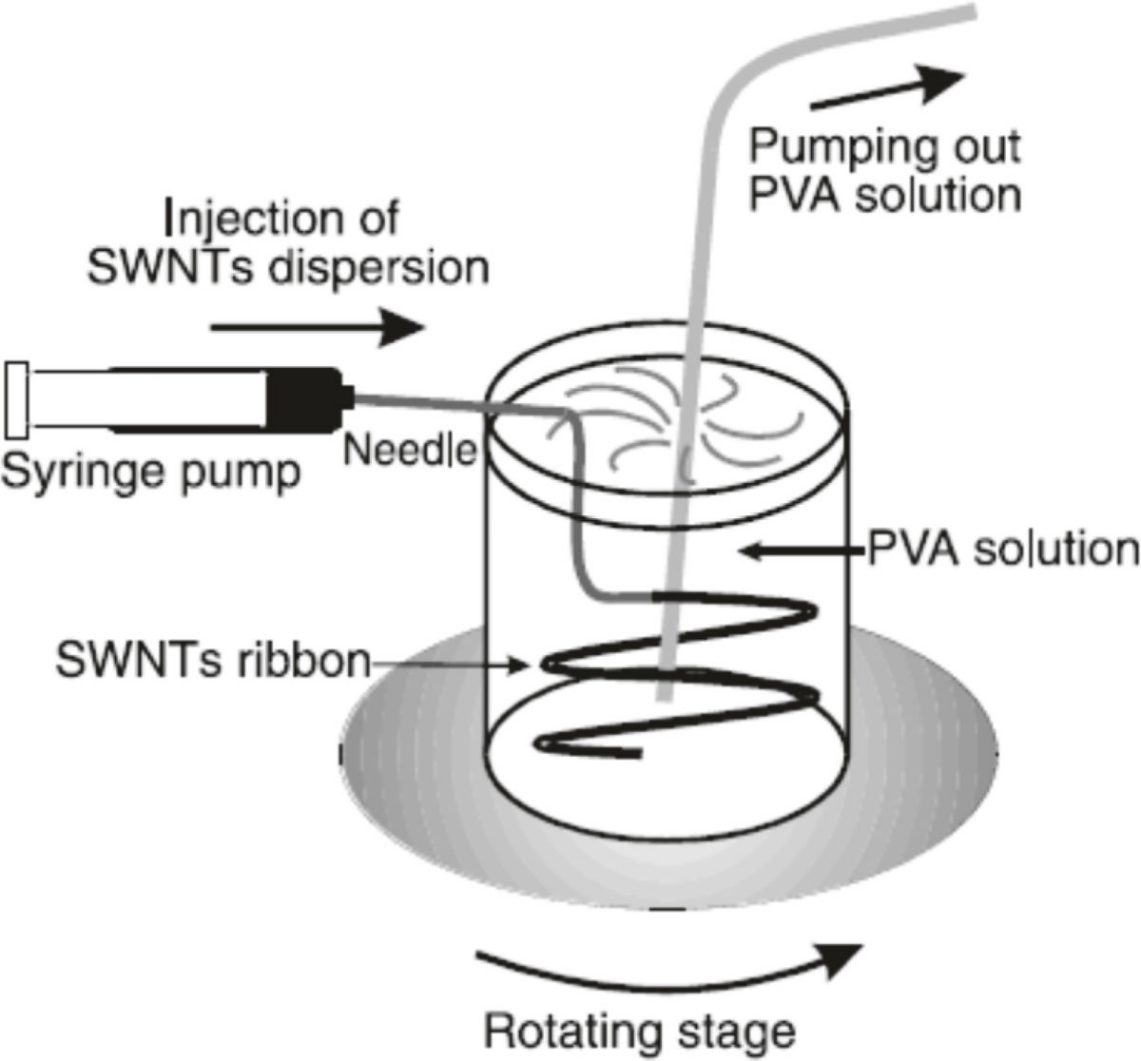
Schematic of wet spinning apparatus setup [29]. Reprinted from [29].

In 2000, a group of French scientists made a breakthrough discovery with the development of carbon nanotube fibers [29]. Carbon nanotube fibers were thin (10–100 microns in diameter) and made of only carbon nanotubes. Carbon nanotubes were sonicated and separated in water using a tissue sonicator with the presence of the surfactant, sodium dodecyl sulfate (SDS). SDS is negatively charged with a long hydrophobic carbon chain. Surfactant separated carbon nanotubes from bundling and aggregating. It adsorbed to the surface of the nanotubes, and the negative charge from the sulfate group produced an electrostatic repulsion of like charges that separated the nanotubes. SWCNT suspensions were then syringed into a 5% aqueous solution of poly(vinyl alcohol) (PVA) that was rotated using a moving stage as shown in Figure 6. Diameters of the fibers could be altered with the use of different flow rates through the syringe and speed (revolutions per minute, rpms) of the rotating stage. Amphiphilic properties of PVA allowed adsorption to the surface of the CNTs and, therefore, displaced the charged SDS surfactant. However, unlike SDS, the neutral PVA cannot provide efficient stabilization against the van der Waals attractive forces of the nanotubes, which causes them to attract and aggregate into ribbons in the aqueous solution. Repeatedly washing the ribbons in water, pulling them out of aqueous solution, and allowing them to dry wash away the excess polymer and surfactant, thus purifying the ribbons into carbon nanotube fibers.

The carbon nanotube fibers displayed outstanding physical and mechanical properties in comparison to traditional carbon fibers. The authors saw Young’s Modulus that was an order of magnitude greater than the modulus of high-quality buckypaper. The fibers also displayed conductivity with a resistivity of 0.1 ohm-cm. Interestingly, when the fibers were analyzed with scanning electron microscopy, the inner shell of the CNT fiber cylinder was found to contain mainly SWCNTs, while the outer layer contained mostly nonconductive sp^3^ hybridized carbonaceous impurities. These carbon impurities were randomly dispersed in the initial ribbons, but moved to the outer edges of the carbon fiber due to capillary action and water evaporation from the original ribbons as they formed fibers.

## 3. Electrospinning of CNT Fibers

The process of electrospinning has also been used to develop carbon nanotube fibers and yarns [76, 77]. Electrospinning involves the applying of a high voltage to a liquid droplet that supercharges the liquid droplet. Electrostatic repulsion counteracts the surface tension, which subsequently stretches the droplet. Once the droplet is stretched and passed a certain critical point, a stream of liquid is known to erupt from the surface, called a Taylor cone. Specific variables that can be varied in electrospinning are molecular weight of the polymer, viscosity of the solution, potential, distance between the capillary and the collection screen, temperature, size of the target, and needle gauge. CNT fibers have been produced by co-electrospinning with polylactic acid (PLA) [78] and polyacrylonitrile (PAN) with HiPCO (high pressure CO) CNTs. The spinning distance was 15 cm and the voltage 25 kV. The fibers were graphitized at 1100°C and then spun into yarns. Raman spectroscopy confirmed the presence of SWNTs in the PLA and PAN matrix. Unfortunately, atomic force microscopy (AFM) and transmission electron microscopy (TEM) images of PAN and PLA nanofibrils display an agglomerated microstructure that accounts for a heterogeneous distribution of SWNTs. This is thought to be detrimental to the mechanical properties and conductivity of the polymer fibers. If clumps of SWNTs are spread randomly throughout the polymer fiber, then this casts doubt on the use of PAN/PLA-CNT fibers as possible electrode materials.

### 3.1. Carbon Nanotube-Polymer Composite Fiber Sensors.

**Figure 7:**
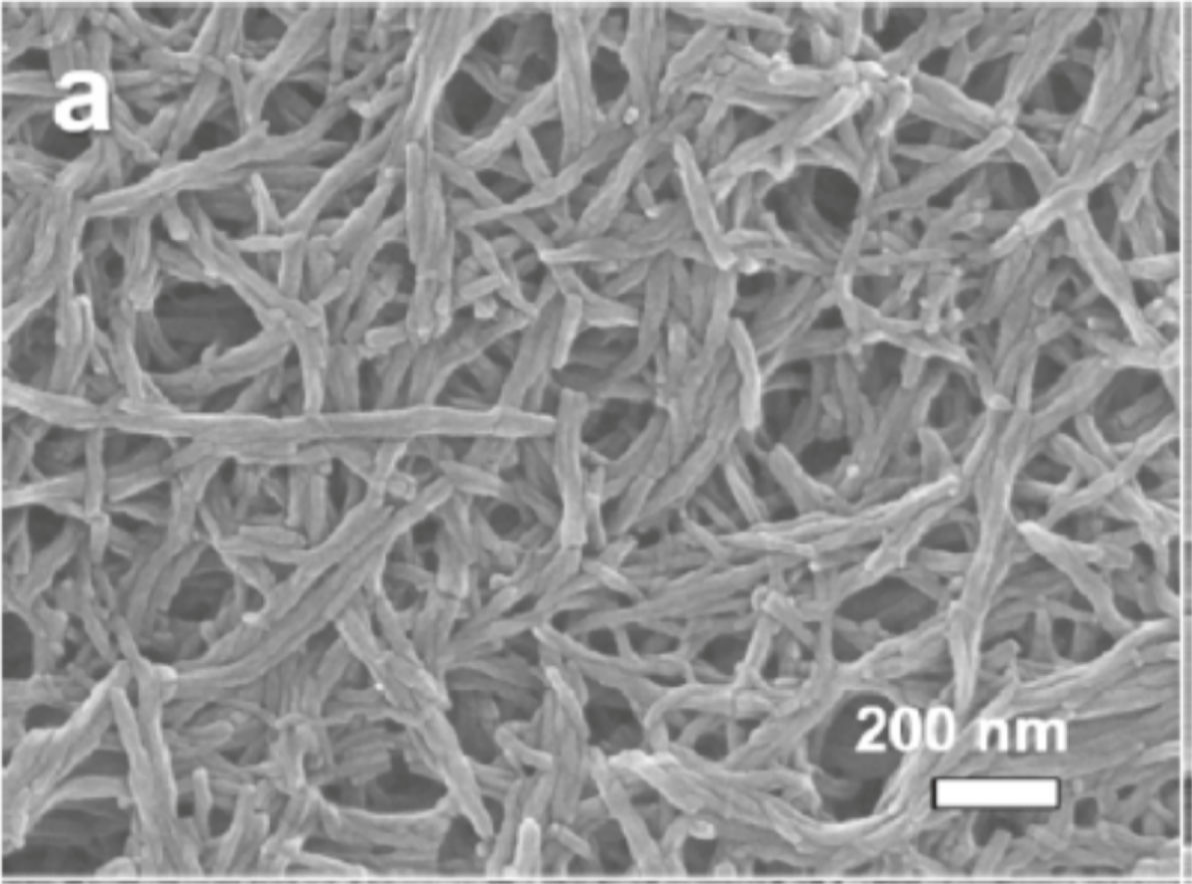
SEM of PANi-CNT fiber [30]. Reprinted from [30].

CNTs have also been used for a wide variety of alternative chemical sensors. Recently, carbon nanotube/polyaniline (PANi) composite nanofibers have been synthesized via a simple polymerization mechanism to produce high performance chemosensors as displayed in Figure 7 [30]. SWCNTs were dispersed in 1.0 M HCl and then aniline plus an aniline dimer initiator was added to form the composite CNT/PANi nanofiber (~20 nm diameter). The conductivity of the nanofibers could be widely tuned (10^−4^ to 10^2^ S/cm) based on varying pH and SWCNT loadings. The great increase in conductivity of the composite fibers can be explained by CNTs interacting with polymer chains that lower the hopping length that exists in pure PANi nanofibers. Electron transfer occurs from the nanotubes to the LUMO of the conjugated polymer, which increases charge carrier mobility. The highly conductive composite nanofibers detected NH_3_ vapor (100 ppb) much quicker than pristine PANi fibers by responding to a 20-fold increase in resistance (120 s versus 1000 s) as shown by the I-V relationships from the four-probe measurement.

### 3.2. Carbon Nanotube Fiber Microelectrodes.

Joseph Wang’s research group was the first to use poly(vinyl alcohol) (PVA) wet-spun carbon nanotube fibers as microelectrodes [79]. The CNT fibers were heat treated at 300°C for sixty minutes and pushed into glass capillaries to form microelectrodes. Scanning electron microscopy (SEM) revealed that heat treated fibers fractured the skin of the fiber, which removed much of the nonconductive poly(vinyl alcohol) polymer and sp^3^ hybridized carbonaceous impurities, while also exposing internal nanofelt porous conducting bundles of nanotubes. Untreated fibers revealed a uniformed porous structure, which mirrored the aligned surface of the CNT. Treated fibers were also found to be more conductive because of their much larger background charging currents in cyclic voltammetry, which is a function of the much larger double layer capacitance. This conductivity is a result of the fact that thermally activated fibers have had their nonconductive carbonaceous and polymer purities removed and because of the highly catalytic ends of the nanotubes (as opposed to the sidewalls) are being exposed at the tips of the fibers. Heat treated carbon nanotube fibers yielded significantly higher current densities for 100 *μ*M dopamine and hydrogen peroxide than carbon fibers and untreated carbon nanotube fibers using hydrodynamic voltammetry. Likewise, chronoamperometric signals were much higher for dopamine and hydrogen peroxide for CNT fibers as opposed to carbon fibers. Hydrodynamic voltammetry measurements for NADH showed that treated CNT fiber electrodes yielded redox activity at potentials above 0.1 V, while redox activity is only observed above 0.6 V for both untreated CNT fibers and for carbon fiber microelectrodes. The electrocatalytic activity of CNT fibers facilitates the minimizing of NADH passivation and hence surface fouling at these lower voltages.

### 3.3. Wet Spinning of Poly(ethyleneimine)- (PEI-) CNT Fibers.

An alternative method was used to wet-spin carbon nanotube fibers in a very similar manner by substituting poly(vinyl alcohol) (PVA) with poly(ethyleneimine) (PEI) [80]. CNT fibers were spun into PEI (40% in MeOH, MW = 40,000) which was used as the coagulant. Three surfactants, cetyltrimethylammonium bromide (CTAB), lithium dodecyl sulfate (LDS), and sodium dodecylbenzenesulfonate (SDBS), were used to separate nanotubes. PEI was found to intercalate into SWCNT bundles that increased conductivity, rigidity, and Young’s Modulus. SEM images showed regions of polymer incorporation that were much less extensive than for PVA-formed CNT fibers. The conductivity of these fibers (100 – 200 S cm^−1^) is 10-fold greater than SWCNTs made from the pulse laser vaporization (PLV) method and 100-fold greater than the PVA wet-spun CNT fibers. The reactive amine group on the PEI polymer interacted with the carbon nanotubes by physisorption to the walls of the nanotubes. The amine group donates a pair of electrons to the SWCNT sidewalls initiating an intermolecular charge transfer, which significantly increases the conductivity of the fiber. Electron dispersive spectroscopy (EDS) showed no presence of sulfur on the surface of the nanotube fibers, which may show that the surfactants (SDBS, LDS, etc.) could have been removed during the coagulation process. A new conductive fiber may prove to be very beneficial for the purpose of using these fibers for electrochemistry.

A new method of constructing epoxy insulated carbon nanotube fiber microelectrodes has been created. CNT fiber microelectrodes were then silver epoxied to a gold pin that fits into the headstage of the potentiostat [39]. Polyethyleneimine carbon nanotube fiber microelectrodes have also been developed for neurotransmitter detection [80]. Previously, PEI-CNT fibers have not been used as electrode materials. The higher conductivity of the PEI-CNT fiber with respect to the PVA-CNT fiber makes these fibers suitable as electrodes for the detection of neurotransmitters using fast scan cyclic voltammetry. A comparison to PVA-CNT fiber microelectrodes, adsorption properties, stabilities in vitro, and a resistance to serotonin and 5-hydroxyindole acetic acid (5-HIAA) fouling was thoroughly examined with these novel PEI-CNT fiber microelectrodes.

### 3.4. Carbon Nanotube Fibers for Neurotransmitter Detection.

Carbon nanotube fibers have further been used to form microelectrodes for small molecule detection using electrochemistry [79]. It has been widely expected that carbon nanotube fibers could be used as neurotransmitter detectors *in vivo*. One of the problems of dopamine detection *in vivo* is that there is often the presence of anionic interferants such as ascorbic acid that are present in concentrations sometimes 100–1000 times higher than dopamine. Furthermore, ascorbic acid is oxidized at similar potentials as dopamine which causes overlapping signals to occur that can prevent the correct concentration estimate of dopamine.

Using differential pulse voltammetry (DPV), carbon nanotube fibers were compared to glassy carbon electrodes (GCEs) in the detection of both dopamine (DA) and ascorbic acid (AA) [81]. At GCEs, dopamine oxidation shifted 80 mV with respect to carbon nanotube fibers but still could not distinguish between dopamine and ascorbic acid. However, current density increased by more than one magnitude at carbon nanotube fiber electrodes compared to GCEs. The amplitude of AA peaks was much lower than that of dopamine, even though it was present at a concentration 10-fold greater than that of dopamine. An electrostatic barrier at the surface of the carbon nanotube fiber could explain this phenomenon. Carbon nanotube fibers contain negatively charged oxide and carboxyl group that electrostatically interact with the positively charged dopamine. However, the anionic AA is negatively charged and is electrostatically repelled by this negative charge, which inhibits adsorption and charge transfer to the negatively charged surface of the CNT fiber. Also, the aromatic group of dopamine undergoes favorable *π-π* interactions with the sp^2^ hybridized CNTs, which further facilitates adsorption to the surface of the carbon nanotube fiber microelectrode. The higher porosity of the CNT fibers also provided further sites for analyte adsorption. dopamine and ascorbic acid were present in equal amounts, the ascorbic acid signal was similar to that of the noise. When ascorbic acid was present at an order of magnitude greater than that of dopamine, the signal for dopamine is still significantly higher than that of ascorbic acid.

**Figure 8:**
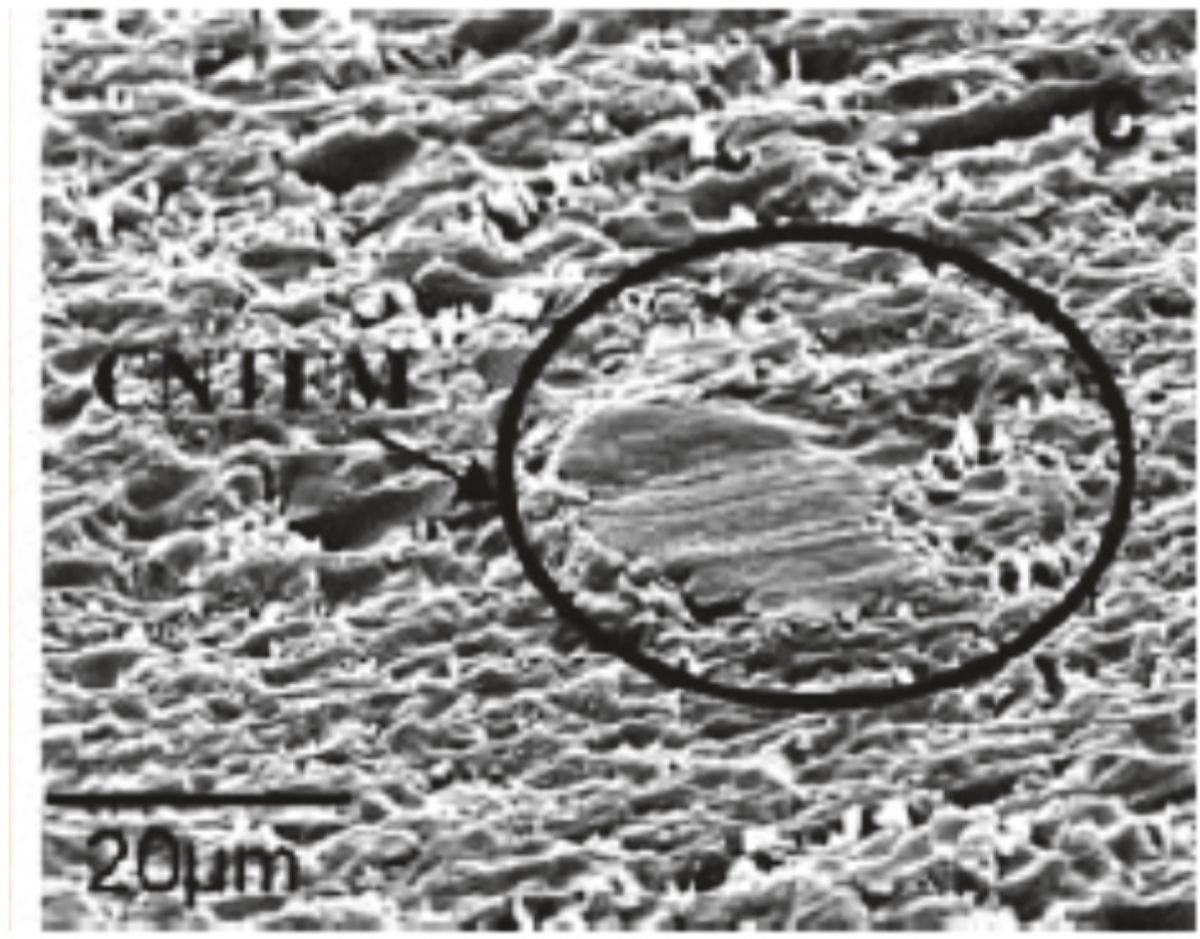
CNT fiber completely sealed by epoxy resin to form CNT-microelectrode [31]. Reprinted with permission from [31].

Viry et al. further developed carbon nanotube fiber microelectrodes for the detection of NADH using cyclic voltammetry and to manufacture glucose biosensors [31, 82]. The glucose sensor was formed by adsorbing a mediator onto the surface of the carbon nanotube fiber. Figure 8 shows the CNT fiber enclosed by epoxy resin to form a CNT fiber microelectrode. At a potential of 0 volts, analytes were oxidized by applying a potential and via the dehydrogenase enzyme. The authors concentrated on several surface modifications for the CNT fibers to make them more sensitive, selective, and responsive to the analytes. They began this procedure by dipping CNT fibers in tetrahydrofuran (THF) and, subsequently, drying to deposit a layer of the insoluble organic molecule on the surface of the CNT fiber. The CNT fibers were hot stretched and pulled to 500% length in a hot flow of air, which produced a substantial increase of CNT alignment along the axis of the fiber. The authors hypothesize that analytes are more likely to adsorb to a well-organized and aligned interface as produced by CNT fiber stretching, which aligns CNTs. The CNT fibers were then dipped into a solution of polyoxometalate (H_3_PMo_12_O_40_) (POM) in 0.5 M sulfuric acid to exfoliate graphite and carbon particles followed by dipping in a buffered solution of pH 8 to desorb the POMs from the surface. The background capacitive charging current increased threefold, which indicates that the electroactive surface area increased substantially due to the increased porosity of the CNT fiber. The authors speculated that the POM layer created new holes in the CNT network via chemisorptions.

Ewing and Safina’s research group utilized wet-spun PVA-CNT fibers to prevent long-term dopamine fouling at the surface of an electrode [70]. Dopamine oxidation occurs by the way of a two-electron transfer, which yields dopamine-ortho-quinone (DOQ). When the amine is protonated in the buffer, cyclization occurs forming leucodopaminechrome (LDC) that further forms dopaminechrome (DC) by undergoing another two-electron oxidation. A melanin polymer is formed from the free radical polymerization of dopaminechrome. Carbon nanotube fibers resisted melanin polymer formation or surface fouling by way of their surface roughness, high electroactivity, fast response, and high chemical stability. The EDX spectra of CNT fibers show traces of nickel (catalyst remaining from CNT synthesis) and higher oxide concentrations with respect to carbon fibers, which explains their higher sensitivity towards dopamine as opposed carbon fibers. For 100 *μ*M dopamine, the CNT fiber electrode displayed more stability against chemical fouling which caused oxidation products of the analyte. After three hours of continuous oxidation, the current decreased by only 15% in CNT fibers, while it decreased by over 70% in carbon fibers.

### 3.5. Carbon Nanotube Yarn Electrodes.

The Sombers research group has recently developed carbon nanotube yarns as microelectrodes for neurotransmitter detection using FSCV. They grew MWCNT arrays on quartz substrates via the “chloride mediated chemical vapor deposition (CVD) method” [83]. They pulled out CNT ribbons from the array and then attached them to a spindle. The yarns were formed by drawing and then rotating the spindle (twist angle 20 degrees). The CNT yarns were then fabricated into disk microelectrodes using a polishing wheel. The yarn electrodes displayed superior selectivity, sensitivity, and spatial resolution in comparison to traditional CFMEs [84]. CVs and background charging currents yielded faster electron transfer kinetics (reduced peak separation, ΔE_p_, for dopamine) and a greater mass transfer profile at the surface of the sensor, which increases sensitivity and substantially lowers the limit of detection in comparison to CFMEs. Signal enhancement (180%) is mainly due to the presence of oxygen functionalized hydroxyl and carboxyl functionalities that electrostatically interact with the positively charged dopamine. A greater cathodic/anodic peak ratio of CNT yarns in comparison to CNTs was observed and could be a function of stronger reversibility of the oxidation to reduction reactions at the CNT surface. CNT yarns also could help resolve multiple analytes in complex mixtures. An extra peak was observed in the voltammogram of adenosine (~ 530 mV), which could help distinguish it from peroxide, histamine, and shifts in pH. The peak is most likely a result of adenosine polymerization onto the surface of the CNT yarn. The yarn electrode also better distinguished dopamine from ascorbic acid, serotonin, and DOPAC. CNT yarn electrodes were further used to detect stimulated dopamine release in brain slices (striatum), which demonstrate their potential applicability as alternatives to traditional carbon fiber microelectrodes.

Carbon nanotube yarns can also be purchased from companies such as Nanocomp Technologies, Inc. and General Nano LLC. They use dry-spinning for the manufacturing of carbon nanotube yarns (CNTYs) as developed by the textile industry [38]. Aligned CNTs are synthesized in the furnace and then twisted to form a yarn. Our lab made carbon nanotube yarn electrodes (CNTYEs) by placing a CNTY in a polyimide coated fused silica capillary that was slipped into a pulled glass capillary that was back filled with 1 M potassium chloride and five-minute epoxy was used to seal the polyimide-glass capillary interface. The electrodes were beveled at an angle perpendicular to the capillary. Background charging currents were found to be 4-fold larger for CNTYEs as opposed to disk CFMEs [38]. In addition to the increase in sensitivity of CNTYEs, dopamine was found to be adsorption controlled to the surface of the CNT yarn as illustrated by concentration and scan rate experiments. As opposed to CFMEs, CNTYEs have an interesting property as their sensitivity for dopamine is independent of the wave application frequency. At lower frequencies, more time is spent at the negative holding potential, which electrostatically attracts the positively charged dopamine. This interesting property of CNTYEs was explained by the fact that rates of desorption of dopamine and dopamine-ortho-quinone (DOQ), the oxidation product, are equal to one at CNTYEs. Higher desorption of DOQ would mean that surface coverage of DOQ decreases, which means that there is less dopamine adsorbed to the surface after reduction for CFMEs. Thus, CNTYMEs could measure changes in neurotransmitters at a much faster timescale, closer to which neurotransmission actually occurs.

### 3.6. Acid Wet-Spun Carbon Nanotube Fibers.

**Figure 9:**
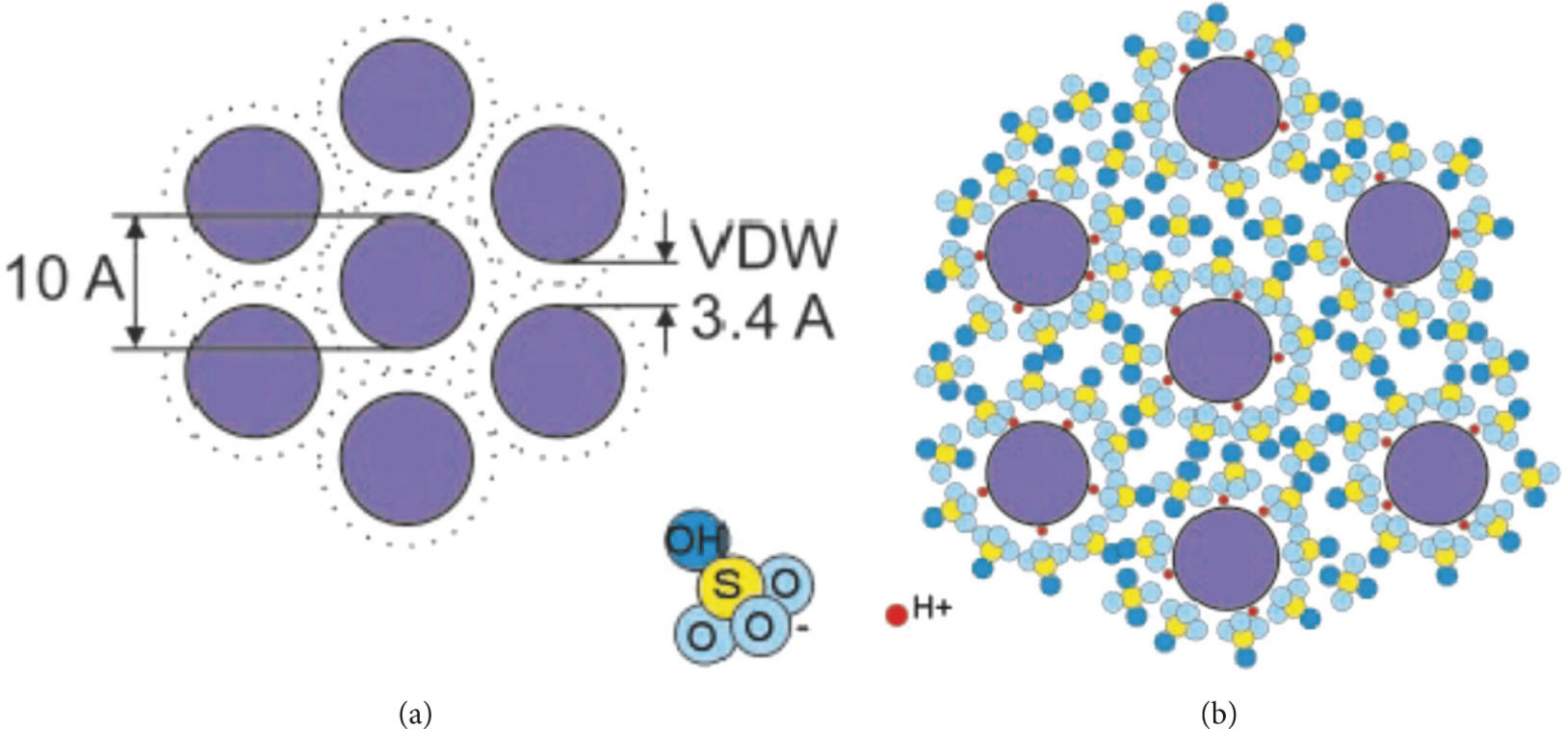
Mechanism of CNT fiber formation. (a) SWNTs in van der Waals contact. (b) SWNT ropes after exposure to sulfuric acid [32]. Reprinted from [32].

Smalley et al. developed a novel method of creating carbon nanotube fibers without the use of polymers or surfactants [32]. They dispersed 8% of SWCNTs in 102% sulfuric acid (including 2% excess sulfur trioxide). The fibers were produced in a custom built apparatus used for mixing and extruded SWCNT fibers. The procedure was similar to wet spinning though the coagulants that were used were diethyl ether, 5 wt% aqueous sulfuric acid, or water instead of the polymer coagulant. Furthermore, no surfactant or sonication was necessary to separate the carbon nanotubes from aggregating via van der Waals interactions. Fibers were dried in a vacuum followed by further annealing in a vacuum at 1100°C. SEMs of the fibers displayed very high vertical alignment of SWCNT ropes that are 20 to 30 nm. Raman ratio peaks of 20:1 (G:D peaks) also confirmed a highly ordered species. Mechanisms of vertical alignment lie in the swelling of the carbon nanotubes by sulfuric acid. Negatively charged anionic oxide groups from the sulfuric acid surround the individual nanotubes forming an energetically favorable charge transfer complex (see Figure 9).

Upon spinning into the coagulant, the anions are displaced by water, which collapses them into vertically aligned carbon nanotube fibers (~ 50 microns in diameter). This process is reversible because the nanotube fibers swollen again into CNT ropes upon subsequent suspension of the fibers into a solution of sulfuric acid. CNT fibers displayed mechanical strength and rigidity much superior to CNT fibers created from the traditional method of polymer wet spinning. The creation of a CNT fiber without the presence of sonication (that cuts nanotubes short and destroys conductivity), polymer, surfactant, and nonconductive carbonaceous impurities provides a potential method of developing highly conductive carbon nanotube fibers that could be used for the purpose of developing microelectrodes for the monitoring of neurotransmission.

Pasquali et al. found that chlorosulfonic acid is the first “true solvent” for carbon nanotubes [33]. Therefore, carbon nanotubes could be dissolved in chlorosulfonic acid without having clumps of differently sized nanotubes suspended in solvents. Wet spinning was performed in the same manner as before, with 2–6% of CNTs dissolved in chlorosulfonic acid spun into a coagulating bath of water or acetone to remove the acid using a spinning chamber in a custom built apparatus to collect fibers on a winding drum. The linear velocity of the drum was higher than the speed of the spinneret to produce high CNT alignment by constant stretching and tensioning of the filament. Four point probes were used to measure conductivity that greatly increased to 5 MS/m prior to iodine doping. Electrical conductivity was 10-fold greater than traditional undoped solid state fibers and 5-fold better than doped (with iodine) CNT fibers. The fibers combined the great conductivity of metals with the specific strength of high performance carbon fibers.

**Figure 10:**
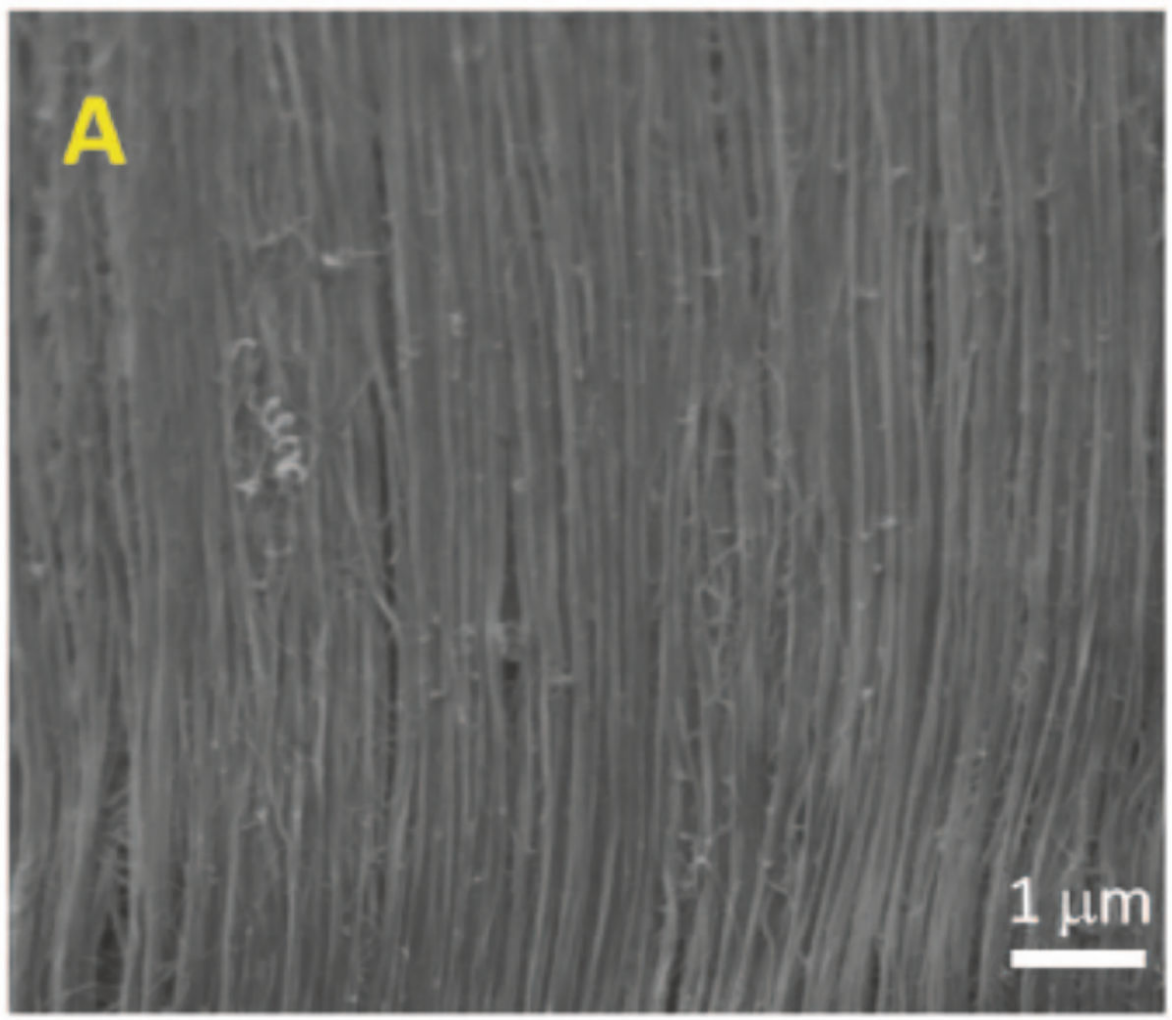
SEM of vertically aligned CNT fiber spun with chlorosulfonic acid [33]. Reprinted from [33].

The aim of this procedure was to fabricate CNTs that are longer and reduce the number of CNT ends in a fiber, producing greater strength and reducing CNT junctions, which, therefore, raise electrical (greater than steel, Ni, and Au) and thermal conductivity (30-fold higher than that of wet-spun fibers). They observed 5 *μ*m long CNT lengths as opposed to 0.5 *μ*m, which greatly enhanced conductivity. As before, the use of chlorosulfonic acid precludes the use of surfactants in the CNT dispersion and polymers to collapse the fibers into ribbons. SEM imaging displayed highly vertically aligned fibers composed of fibrils that were 10 to 100 nm in diameter (see Figure 10). Highly aligned fibril structures made the fibers ultraconductive due to increased CNT overlap. Decreased spacing between the highly aligned fibrils facilitated inter-CNT transport. Iodine doping of the fibers further increased conductivity (~5- to 10-fold) due to the increase in intra-CNT conductivity of the semiconducting CNTs and also increasing disorder, which helps relax the momentum conservation need for inter-CNT transport. The iodine fibers were so conductive and strong that they could support and conduct electricity to light a light-emitting diode lamp and also created a field-emitting device displaying metallic field emission [33].

Furthermore, chlorosulfonic acid spun CNT fibers have been developed as electrode materials for neurotransmitter detection using fast scan cyclic voltammetry. The use of acid spun CNT fibers is promising for a few reasons. First of all, the high conductivity of the CNT fibers makes it desirable as an electrode material for voltammetry. Second, the CNT fiber formation precludes the use of polymers and surfactant that coat the CNT surface (such as poly(vinyl alcohol) and sodium dodecyl sulfate) that coat the CNT surface, which decrease adsorption by blocking adsorption sites for biomolecules. Third, this method does not include the use of sonication to separate CNT bundles, which decreases CNT length and lowers conductivity. Finally, the use of the acid could oxidize the surface groups of the CNT fiber with negatively charged oxide groups that would make the electrode material electrostatically attract the cationic dopamine.

### 3.7. Growth of Vertically Aligned CNTs Using Chemical Vapor Deposition.

The growth of carbon nanotube yarns using chemical vapor deposition could be potentially useful for neurotransmitter detection [83]. A novel method depicts a one-step growth of aligned bulk carbon nanotubes by chloride mediated chemical vapor deposition. Using a quartz substrate, an iron chloride catalyst was utilized along with acetylene to grow vertically aligned carbon nanotubes at 820°C at 10 Torr. Growth occurred over a time period of approximately 20 minutes to produce 2.1 mm length of vertically aligned CNTs. It was found that growth was highly dependent on both temperature and pressure. The vertically aligned CNTs were then spun into yarns. The mechanism of growth is that iron chloride reacts with acetylene to produce carbon-rich iron carbide (FeC_2_) and hydrogen chloride (HCl). Iron carbide then segregates into graphene layers, which initiates CNT growth in chloride mediated chemical vapor deposition (CM-CVD). Growth is triggered once budlike CNT structures are formed. This one-step CM-CVD growth of vertically aligned carbon nanotubes that can be spun into yarns is a promising method of producing CNT yarns that could be used as sensors.

The Meyyappan group has pioneered growing carbon nanotubes and nanofibers using plasma enhanced chemical vapor deposition (PECVD) [85]. PECVD differs from CVD in that it includes a plasma source which arises from an alternating or direct current discharge between two electrodes. It is not currently known what the role of the plasma is in carbon growth or CNT growth is, but empirical evidence suggests that the electric field in the plasma enables vertically aligned CNTs or carbon than thermal CVD [86].

## 4. Carbon Nanofiber Electrode Array Development Using Plasma Enhanced Chemical Vapor Deposition

Carbon nanofiber (CNF) arrays grown using plasma enhanced chemical vapor deposition have been developed by scientists at Oak Ridge National Laboratory for many electroanalytical measurements [87]. A thin layer of metal catalyst was deposited onto a silicon wafer to begin the process. The fiber synthesis was performed using direct current-plasma enhanced chemical vapor deposition (PECVD) where the decomposition of acetylene on the nickel results in “tip-type” nanofibers. The CNF array was used as an electrode array where excitable cell matrices of both neuronal-like derived cell lines (PC-12) and primary cells (from the rat hippocampus) were cultured. The electrode arrays detected dopamine, norepinephrine, and 5-hydroxytyramide using linear sweep voltammetry, amperometry, and cyclic voltammetry over a 16-day period. The same group also used these vertically aligned carbon nanotube arrays for stimulation and extracellular recording of spontaneous and evoked neuroelectrical activity in organotypic hippocampal slice cultures [88] and as a neural chip that stimulated and monitored electrophysiological signals from brain tissue *in vivo* [89].

Carbon nanofiber electrode arrays have also been used with fast scan cyclic voltammetry for neurotransmitter detection [90]. CNFs were prepared as arrays on silicon wafers as 3 x 3 electrode pads. Using plasma enhanced chemical vapor deposition (PECVD), vertically aligned CNFs were grown using ethylene feedstock and nickel catalysts at 700°C. Wireless Instantaneous Neurotransmitter Concentration Systems (WINCS) were used for the data analysis software to integrate FSCV and digital telemetry. Dopamine was detected in concentrations spanning from 500 nM to 2.5 *μ*M. Dopamine detection was modeled and compared to CFME detection by taking into account measurements of the electroactive surface areas. CFMEs and CNTs were found comparable in their response and sensitivity to dopamine detection, which illustrates the applicability of CNF arrays as electrodes to be used for neurochemical monitoring.

The growth of edge plane graphene using plasma enhanced chemical vapor deposition onto metal wires was also achieved [90, 91]. This method of carbon growth is attractive for many reasons. First, it does not require the deposition of a metal catalyst onto the surface of the metal wire, which is tedious and often difficult to achieve as opposed to the growth of carbon nanotubes [91, 92]. Second, it also has uniform surface coverage over the entire metal. This is important to create carbon coated metal electrodes that will be used for neurotransmitter detection using voltammetry. Metal is used as the substrate for its fast electron transfer kinetics; however, it must be coated with carbon since metal electrodes have a different overpotential for the oxidation of water that could interfere with the signal during voltammetry.

## 5. Advantages and Disadvantages of Electrochemical Techniques

Some methods used to measure neurotransmitters such as dopamine in addition to carbon fiber micro/nanoelectrodes include positron emission tomography (PET), genetically encoded fluorescent sensors, and microdialysis. Carbon electrodes can be used to detect dopamine on a relatively fast time scale, but they often cannot detect basal levels of neurochemicals or many analytes simultaneously. PET noninvasively measures dopamine through receptor binding. The major disadvantage of this method is the high cost and lack of widespread availability among the scientific community, especially preclinical scientists [93]. Genetically engineered fluorescent probes are also a relatively new *in vivo* measurement method and are also not widely available [94]. Microdialysis has also become a powerful technique utilized to measure dopaminergic neurotransmission [95]. During microdialysis, buffer is perfused into a specific brain region sampling the extracellular synapse, collected, and then analyzed. The probe that samples a specific brain region is ensheathed in a semipermeable membrane that is permeable to dopamine and other neurotransmitters, but not proteins. Microdialysis probes are relatively large, though, and can cause harmful tissue damage and elicit immune response [96]. They require techniques such as capillary electrophoresis [97], liquid chromatography [98], or mass spectrometry [99] for detection at relatively slow time scales 3–20 minutes [100].

The inherent advantage of carbon electrodes are that they (1) are small (micron-nanometer scale) [101], (2) are biocompatible [1], and (3) have fast electron transfer kinetics, conducive to making fast measurements *in vivo* [26]. The small size of the electrode (usually 7,000 nm in diameter or less) allows for the accurate target of brain regions such as the core of the nucleus accumbens or prefrontal cortex that are rich in dopaminergic neurons and dopamine [102]. On the contrary, microdialysis probes have much larger steel shaft diameter up to .5 mm. Furthermore, it limits tissue damage and can target small brain regions as in small model organisms such as the fruit fly [1]. As their name suggests, “carbon” electrodes are biocompatible for implantation in model organisms or humans [26] without causing tissue damage or eliciting an immune response [102]. As opposed to metal microelectrodes, carbon electrodes also have a lower overpotential for the oxidation of water, which allows for scanning at higher voltages. Lastly, as opposed to the aforementioned techniques, carbon electrodes, when used with voltammetry, measure fast subsecond changes in neurochemical concentrations. This is incredibly useful for measuring the phasic firing of dopaminergic neurons *in vivo*, which release and uptake dopamine on a fast subsecond timescale.

## 6. Comparison of Limits of Detection and Charge Transfer

As shown in Table 1, a plethora of different carbon electrodes exist. They are thoroughly compared with respect to their carbon material type, type of electrochemical detection, and limit of detection [19]. It is impossible to directly compare each carbon electrode to the other due to differences in electrode size (diameter, shape, and morphology), method of electrode construction (i.e., glass, polyimide coated capillary insulations, etc.), and method of electrochemical detection (i.e., amperometry, differential pulse voltammetry, linear sweep voltammetry, fast scan cyclic voltammetry (FSCV), etc.). Therefore, this comparison will be a qualitative comparison rather than a quantitative comparison. Furthermore, there are also different methods of calculating the limits of detection such as different comparisons to the signal to noise ratio (S/N) and other methods of limits of quantification. On average, carbon nanotube-based sensors have the lowest limits of detection due to their larger electroactive surface areas, fast electron transfer kinetics, and a resistance to surface fouling when utilized with fast scan cyclic voltammetry [19]. Graphene based sensors, n-doped carbon-based sensors, and polymer coated based sensors all tend to have nonconductive impurities that hinder dopamine adsorption [19] and hence do not have low limits of detection. For example, boron doped diamond electrodes resist surface fouling, but often have poor limits of detection due to the relatively low conductivity of the diamond substrate, even when doped with boron [20]. Carbon nanotube microelectrodes also have higher sensitivities for the catecholamine dopamine than for the negatively charged uric acid and ascorbic acid. CNTs are functionalized with negatively charged oxide groups that electrostatically repel the anionic uric acid and ascorbic acid. These negatively charged oxide groups also allow for electrostatic attraction of the positively charged catecholamine dopamine, thus enabling dopamine adsorption to the surface of the electrode increasing sensitivity and, hence, having lower limits of detection.

## 7. Effects on Sensitivity and Charge Transfer with Dopamine

Table 2 shows the diameters and conductivities of different types of carbon fibers utilized as electrode materials for the detection of neurotransmitters using fast scan cyclic voltammetry. PAN-base fibers are formed by graphitizing poly(acrylonitrile) at extremely high temperatures, over 1000 K, which causes the carbon to become more sp^2^ hybridized and hence more conductive. On the other hand, pitch-based fibers are a byproduct of petroleum processing which contain heteroatoms after carbonization. When comparing the more conductive HM to the T-650 carbon fiber, the less conductive T-650 has a significantly higher peak oxidative current for dopamine and higher ΔE_p_, indicating slower electron transfer kinetics. However, the opposite is true for anions DOPAC (3,4 dihydroxyphenylacetic acid) and ascorbic acid, where the more conductive HM fiber electrodes have higher ΔE_p_ values and higher peak oxidation current for the anions. This could be explained by the positively charged catecholamine kinetics being adsorption controlled onto the surface of the carbon fiber, while the kinetics for the anions (DOPAC and ascorbic acid) being diffusion controlled. Since kinetics are also controlled by surface processes, the negatively charged oxide groups on the surface of the carbon fiber would electrostatically repel the anions, hence leading to slower kinetics. Therefore, the type and conductivity of the fiber would play an important role in differentiating dopamine and other catecholamines from their anionic interferants.

## 8. Detection of Dopamine Levels in Presence of Interferants

**Figure 11:**
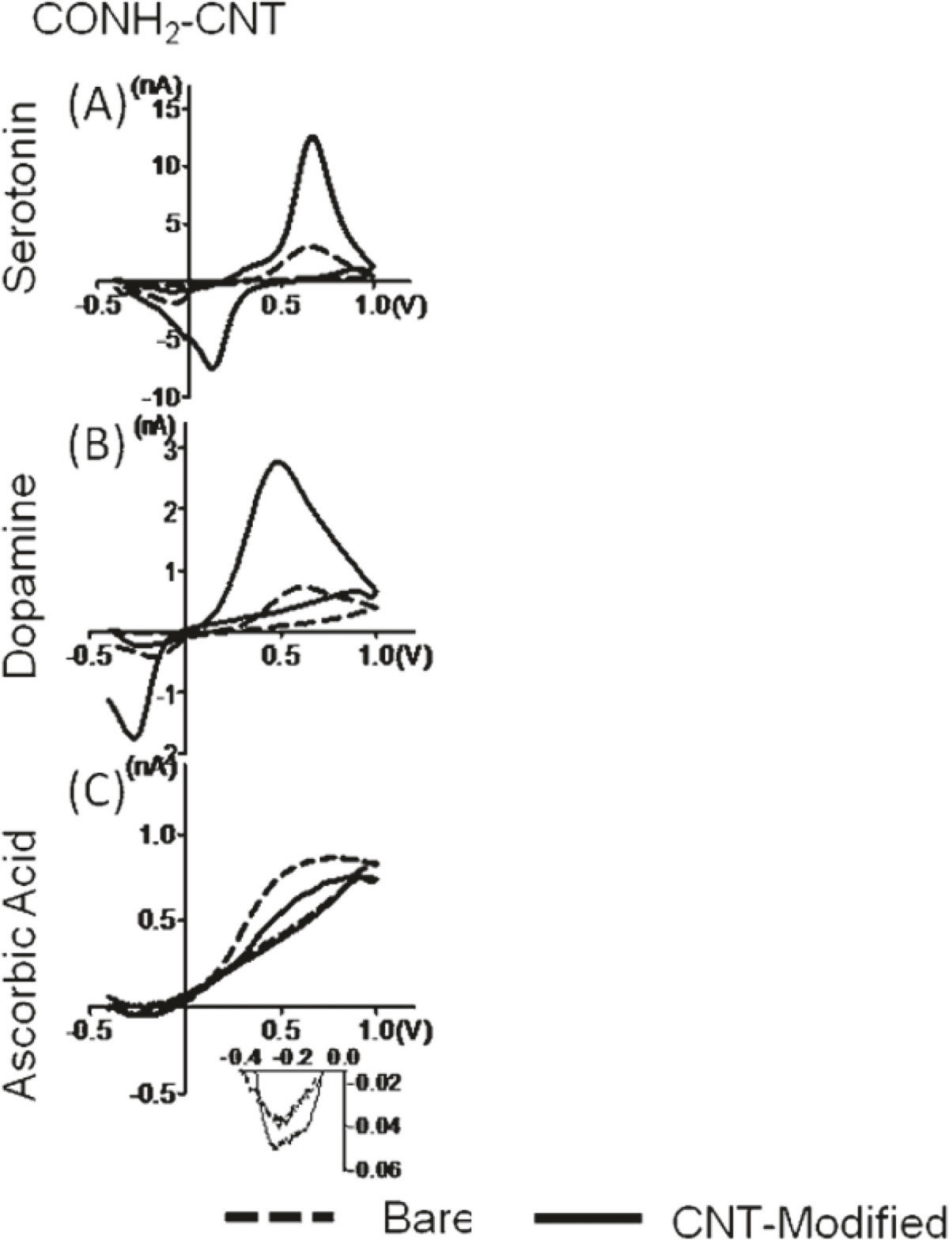
Representative cyclic voltammograms from electrodes before (dashed lines) and after modification with carbon nanotubes (solid line). Vertical columns compare types of functionalized nanotubes: amide-CNTs (panels (A)–(C)), carboxylic acid-CNTs. Figure reproduced with permission from [16].

Carbon nanoelectrodes also enjoy superior sensitivity for dopamine over other interferants such as ascorbic acid and uric acid. Furthermore, dip-coating carbon nanotube functionalized groups onto the surface of the carbon fiber can further enhance dopamine selectivity over anionic interferants. Using FSCV, the negative holding potential allows for the adsorption and preconcentration of the positively charged dopamine and serotonin on the surface of the carbon fiber microelectrode. On the other hand, the negative holding repels the negatively charged ascorbic acid, which is diffusion controlled onto the surface of the electrode rather than adsorption controlled. Modifying CFMEs with amide functionalized carbon nanotubes further enhance selectivity for dopamine and serotonin over ascorbic acid as shown in Figure 11. It is hypothesized that the negative charge density from the oxygen in the carbonyl of the amide further facilitates this selectivity by causing the electrostatic repulsion of ascorbic acid and allowing for more dopamine and serotonin adsorption to occur on the surface of the carbon fiber microelectrodes.

## 9. Physicochemical and Electrochemical Differences

Furthermore, several physicochemical properties lead to their electrochemical performance. Metal microelectrodes have been often seen as poor sensors for dopamine because they oxidize water when scanned to high potentials, which can cause a lot of signal interference. Also, metal microelectrodes are not as easily functionalized with oxide groups as are carbon fiber microelectrodes, which does not allow for dopamine adsorption and preconcentration at the surface as occurs with carbon fiber microelectrodes. Scanning a carbon electrode (polyacrylonitrile pyrolyzed to loosely ordered sheets of graphene) to 1.3 V can renew the surface of the carbon electrode to prevent surface fouling where a polymer forms at the surface of the electrode, hence decreasing sensitivity. When scanning to 1.3 V, carbon-carbon bonds are broken hence increasing the surface roughness of the electrode, which provides for more sites for dopamine adsorption and sensitivity. Also, scanning to 1.3 V functionalizes the surface of the electrode with negatively charged oxide groups that electrostatically attract positively charged catecholamines such as dopamine which increase sensitivity for neurotransmitter detection. For these reasons, even though metals are more conductive than carbon fibers, carbon fibers have been used as the standard electrode material for neurotransmitter detection using fast scan cyclic voltammetry.

Even between different types of carbon electrodes, there are many differences in surface morphology. Carbon fibers, for example, are formed from pyrolyzing polyacrylonitrile into loosely ordered sheets of graphene. The surface is relatively smooth and uniform and fouls easily with neurotransmitters such as serotonin. On the other hand, carbon nanotubes are formed from arc discharge. Carbon nanotubes are highly sp^2^ hybridized with delocalized electrode to make them sufficiently conductive to serve as an electrode material. The cylindrical structure gives them high surface areas and aspect ratios, which is ideal for neurotransmitter adsorption and oxidation. They also display a higher ratio of edge plane carbon over basal plane carbon that contains dangling sp^3^ hybridized carbons that can be easily functionalized with negatively charged oxide groups which allows for dopamine adsorption and increases sensitivity. The increases in surface roughness of the carbon nanotubes also prevent the formation of polymers and fouling at the surface of the electrode.

A novel physical property of the carbon nanotube electrode is that it has a response towards dopamine that is independent of the applied wave application frequency. Small crevices exist on the surface of the carbon nanotube fiber which have been shown to trap dopamine. The desorption of dopamine-orthoquinone has been shown to be much faster at CFMEs than on CNT-microelectrodes. When scanning at 2,000 V/sec and at 500 Hz, a 2 msec temporal resolution has been achieved. These electrodes may be able to detect dopamine at the time scale of the phasic firing of dopaminergic neurons [91].

## 10. Recent Advances and Future Directions in Carbon Nanoelectrodes

Recent advances [19] in the development of carbon nanotube fiber microelectrodes include the growth of carbon nanospikes [90] and carbon nanotubes [91] on metal wires for the detection of dopamine. The uniform growth of carbon allows for the development of a selective and sensitive sensor for dopamine, while maintaining a conductive metal core conducive to electron transfer. Laser treating carbon nanotube yarn microelectrodes increases the surface area and oxygen containing functional groups that allowed for more dopamine adsorption to occur [103]. O_2_ plasma etching and antistatic gun treatment increases dopamine sensitivity 12-fold by increasing oxide functional groups and surface roughness on the surface of the carbon nanotube yarn, respectively [104]. Moreover, crevices on the surface of carbon nanotube yarns and fibers allowed for the trapping of dopamine, which produce thin layer conditions and a response that is independent of the wave application frequency [105]. This remarkable property could potentially allow for the rapid detection of dopamine during fast phasic firing of dopaminergic neurons at 2msec temporal resolution when utilized with higher scan rates.

Aside from dopamine, recent advances using carbon electrodes have examined the detection of several other biologically relevant compounds [106–108]. The Sombers group alone has created carbon electrodes for the detection of dopamine [108], enkephalins [109], glucose [110], and hydrogen peroxide [111–113]. The Wightman group has also used fast scan cyclic voltammetry to the reduction of oxygen, which is important for cerebral blood flow [114–116]. Moreover, the Hashemi group has also focused on the measurement of histamine [117, 118], serotonin [119], and metals [120] using carbon electrodes. Comparable studies also exist for the detection of adenosine [121–124] and ATP [125]. As more advances in the development of carbon electrodes are made, further applications will be available for the sensitive and selective measurement of multiple neurotransmitters in vitro and *in vivo* that are currently available only with microdialysis with LC-MS/MS for detection [126, 127]. The development of carbon electrodes will provide many opportunities for future work for the detection and measurement of multiple neurochemicals that could correlate to specific behavioral, disease, or pharmacological states to solve complex problems in the neurosciences.

This literature review provided a broad overview of the alternative insulations and carbon nano-based microelectrodes that could be utilized for the monitoring of neurotransmitters. This review has examined several different methods of electrode development to produce alternative electrodes to the traditional glass capillary CFMEs including a wide array of carbon nanoelectrodes. The development of novel carbon nanoelectrodes will enhance neurotransmitter detection and allow scientists to better correlate neurochemical measurements to behavior to understand disease states such as drug abuse, Parkinson’s disease, depression, bipolar disorder, and other disease states.

## Figures and Tables

**Table 1:** Carbon nanomaterial-based electrochemical sensors for detection of dopamine, ascorbic acid, and uric acid. The table is reproduced with permission from [19].

Sensor	Method	Analyte	LOD
**Carbon nanotube-based sensors**			
CONH_2_-CNT/CFME	FSCV	DA	0.13 *μ*M
COOH-CNT/CFME	FSCV	DA	0.18 *μ*M
SWCNT forest/CFMEs	FSCV	DA	0.017 *μ*M
Helical CNTs GCE	Differential Pulse Voltammetry (DPV)	DA	0.8 *μ*M
		AA	0.92 *μ*M
		UA	1.5 *μ*M
PDDA/Helical CNT/GCE	DPV	DA	0.08 *μ*M
		AA	0.12 *μ*M
		UA	0.22 *μ*M
CNT yarn disk electrode	FSCV	DA	0.021 *μ*M
CNTYMEs	FSCV	DA	0.01 *μ*M
CNF/GCE	DPV	DA	0.05 *μ*M
s-SWCNT/PET	FET	DA	10^−12^ *μ*M
**Graphene based sensors**			
Craphite oxide bulk/CPE	DPV	DA	0.015 *μ*M
		UA	2.7 *μ*M
Graphene flower/CFE	DPV	DA	0.5 *μ*M
		AA	24.7 *μ*M
		UA	2 *μ*M
3D graphene foam electrode	Amperometry	DA	0.025 *μ*M
SWCNH/GCE	LSV	DA	0.06 *μ*M
		AA	5 *μ*M
		UA	0.02 *μ*M
Whole Graphene solution-gated graphene transistor	SGGT	DA	0.001 *μ*M
		AA	0.01 *μ*M
		UA	0.03 *μ*M
**N-doped carbon based sensors**			
N-doped graphene/SPCE	CV	DA	0.93 *μ*M
N-CNRs-Nafion/GCE	DPV	DA	0.0089 *μ*M
N-PCNPs/GCE	DPV	DA	0.011 *μ*M
		AA	0.74 *μ*M
		UA	0.021 *μ*M
**Polymer coatings**			
PEDOT/RGO/GCE	Amperometry	DA	0.039 *μ*M
PEDOT/CNT/CPE	DPV	DA	0.020 *μ*M
PEI/CNT/CFME	FSCV	DA	0.005 *μ*M
Gr/(PDDA–[PSS-MWCNTs])_s_ graphite electrode, LBL	Amperometry	DA	0.15 *μ*M
CNP/functionalized silicate particles/ITO, LBL	DPV	DA	0.125 *μ*M
PANANA–MIPs/GCE	DPV	DA	0.0033 *μ*M
MIPs-Craphene/GCE	DPV	DA	10^−5^ *μ*M
PPy/CNTs-MIPs/GCE	DPV	DA	10^−5^ *μ*M
**Indirect detection using enzymes and DNA**			
Cysteamine/MWCNT–tyrosine–Nafion/Au electrode biosensor	Amperometry	DA	0.003 *μ*M
	DPV	DA	0.05 *μ*M
Uricase/Chitosan/CNT nanofiber/AgNP/Au electrode biosensor	Amperometry	UA	1 *μ*M

**Table 2:** Conductivity and diameter of carbon fibers. The table is reproduced with permission from [9].

Fiber	PAN-based Conductivity (nS/m)	Diameter (*μ*m)	Fiber	Pitch-based Conductivity (nS/m)	Diameter (*μ*m)
T-650 [a]	67	7	P-25 [a]	77	11
HM [b]	111	8	P-55 [a]	118	10

[a] Product information from Cytec Industries.

[b] Product information from Goodfellow corporation.
